# Test-retest reliability of MEG functional brain connectivity related to language production: Behavioral, functional, and structural underpinnings of reliable connectivity

**DOI:** 10.1162/imag_a_00550

**Published:** 2025-04-25

**Authors:** Heidi Ala-Salomäki, Marijn van Vliet, Jan Kujala, Timo Roine, Mia Liljeström, Riitta Salmelin

**Affiliations:** Department of Neuroscience and Biomedical Engineering, Aalto University, Aalto, Finland; Aalto NeuroImaging, Aalto University, Aalto, Finland; Department of Psychology, University of Jyväskylä, Jyväskylä, Finland; BioMag Laboratory, HUS Medical Imaging Center, Helsinki University Hospital, Helsinki, Finland

**Keywords:** functional connectivity, structural connectivity, magnetoencephalography, picture naming

## Abstract

The number of studies examining functional connectivity of the human brain is increasing rapidly. In this magnetoencephalography (MEG) study, we examined the reliability of connectivity related to language production in a picture naming test-retest paradigm, using data collected from the same participants on 2 separate days. We determined the connections that were reliable (Intraclass Correlation Coefficient, ICC) across both days and also examined the behavioral, functional, and structural properties underlying this reliability. A particularly salient finding among a rich set of results was a reliable pattern of beta connectivity increase in the left motor and frontal regions (0–400 ms and 400–800 ms after picture onset) and gamma connectivity decrease in the bilateral motor regions (800–1200 ms) which we suggest to represent the motor preparation of speech production. Furthermore, the reliable connections tended to be more frequently associated with language performance than the non-reliable ones. Finally, the reliable connections were also linked to stronger functional connectivity, as well as to stronger structural connectivity and shorter structural path length, as determined through diffusion MRI (magnetic resonance imaging). Overall, this study defines reliable language production-related functional connectivity and introduces practices that may increase reliability.

## 1 Introduction

The use of functional connectivity metrics in neuroscience research is rapidly growing, which has also highlighted the importance of the reliability and validity of those metrics (see e.g.,Noble et al., 2019). Test-retest reliability refers to the consistency of a measure across repeated measurement sessions, and validity refers to the association of a measure with a defined process. In this study, we sought to determine the reliability of magnetoencephalography (MEG) functional connections in a test-retest scenario involving a language production task. A further aim was to define factors affecting reliability, such as the validity of the findings.

Many studies of functional connectivity are performed on resting-state data. Resting state refers to the brain’s intrinsic and spontaneous activation in the absence of an external task. However, rest may not be the optimal condition for obtaining reliable connectivity estimates, since its unconstrained nature can lead to non-stationary signal patterns due to, for example, arousal and movement (Cohen, 2018;Elton & Gao, 2015;Finn et al., 2017;Greene et al., 2018;Noble et al., 2019), which may cause spurious connections that do not replicate in a test-retest scenario (reviewed inFinn et al., 2017;Noble et al., 2019). Furthermore, resting-state connectivity analysis typically provides a static view of the mean connectivity patterns over time. Yet, functional connectivity is thought to reconfigure in meaningful ways across time and task states (Cohen, 2018;Elton & Gao, 2015;Medaglia et al., 2015). Hence, we examined the test-retest reliability during a picture naming task, which is a constrained, yet engaging language task that requires the interaction between multiple neural systems. By contrasting the picture naming task with a simple visual task, we aimed to highlight language production-related functional connectivity.

For language production, the connectivity of the motor cortex seems to play an important role. During picture naming, the connectivity between the supramarginal gyrus or posterior middle temporal gyrus and (pre-)supplementary motor area, insula, and mid-cingulate has been suggested to be related to the preparation for language production (Liljeström et al., 2015). Also, during silent verb generation the connectivity of the left premotor area has been linked with language production (Li et al., 2020). Despite these convergent findings, the functional connections for language production remain largely unknown and may reach outside of areas typically associated with language processing. In earlier connectivity studies on language, the focus has typically been on a limited selection of activation-derived or user-defined brain regions or restricted exclusively to the left hemisphere (see e.g.,Schoffelen et al., 2017). For a comprehensive description of the connectivity patterns related to language production, we chose to widen the spatial perspective to include both hemispheres and also brain regions that are not commonly reported in language studies.

In addition to determining the reliability of the connectivity estimation, the validity of the observed connectivity patterns needs to be verified. The validity of a finding means that the finding corresponds to the brain process we are claiming to measure. However, often the validity of the finding is not even considered or discussed when studying reliability. This consideration is especially lacking when studying connectivity during resting state, a paradigm frequently used for drawing conclusions about brain networks and their reliability. One approach to validate the functional connectivity patterns is to relate them to behavior, since connections linked to behavior are likely to represent task-relevant brain processes (Cohen, 2018;Jiang et al., 2020). Therefore, an increasing number of studies have recently begun to investigate the association between functional connectivity and behavioral performance. Indeed, there are results showing that the modulation of functional connectivity is associated with behavioral performance (Bassett & Mattar, 2017;Elton & Gao, 2015;Finn et al., 2015;Jiang et al., 2020). Interestingly, both resting-state studies (Mollo et al., 2016;Vatansever et al., 2017;Yu et al., 2018) and a study investigating connectivity during language tasks (Tomasi & Volkow, 2020) have demonstrated the importance of frontal connections for language performance. Critically, there is a lack of research systematically studying how the behavioral relevance of the findings intertwines with the reliability of the findings (but see,Noble et al., 2017). In this study, we investigated whether the observed reliable connectivity was linked to the subjects’ behavior.

When examining the reliability of functional connectivity, it is important to note that multiple factors may influence the reliability of the observed connectivity patterns. There is evidence that the functional and structural properties of the brain affect the reliability of functional connectivity. Earlier findings suggest that the strength of a resting-state functional connection predicts its reliability (Garcés et al., 2016;Noble et al., 2019). There is also some evidence that structurally connected brain regions have more reliable resting-state functional connections than structurally unconnected ones in humans (Griffa et al., 2017) and macaques (Shen et al., 2015). Accordingly, we aimed to gather insights into the functional and structural properties underlying reliable functional connections.

To determine reliable functional connections related to language production, we used a picture naming test-retest paradigm. Within the picture naming task, we focused on the processing stages prior to overt language production. We combined a well-controlled study design with a data-driven approach to select interareal connections for reliability analysis. We also tested the impact of the selection criterion on the results. The field of neuroimaging has only recently begun to investigate the factors affecting reliability of functional connectivity (see for a reviewNoble et al., 2019). This study makes a critical leap by studying whether the reliability of the findings is linked with the behavioral relevance of the findings. Moreover, this study aimed to expand the current limited knowledge on functional and structural properties behind the reliable functional connections.

## 2 Methods

### 2.1 Participants

Twenty healthy human participants took part in the MEG measurements (10 females and 10 males; mean age 25 years; SD 3.9; age range 21–35 years). MEG data were recorded in two separate measurement sessions (1–13 days apart, mean 4.2, SD 3.9). All participants were native Finnish speakers, right-handed, had no history of neurological, psychiatric, or developmental disorders or learning disabilities, and had normal or corrected-to-normal vision. One participant’s data were not used for the analysis due to non-compliance with the MEG task instruction. In addition, one participant was excluded from the analysis of the behavioral data due to non-compliance with the behavioral task and another participant from the analysis of structural data due to preprocessing difficulties. Written informed consent was obtained from all participants, in agreement with the prior approval of the Aalto University Research Ethics Committee. The MEG evoked responses and oscillatory activity of this data set have previously been analyzed inAla-Salomäki et al. (2021).

### 2.2 Experimental design

#### 2.2.1 Behavioral tasks

Outside the MEG device, the participants performed the following behavioral tasks: rapid automatized naming (RAN) (Denckla & Rudel, 1976) and rapid alternating stimulus (RAS) (Wolf, 1986) estimating naming speed, as well as category fluency (animal) and letter fluency (s-letter) (Benton, 1968) estimating word generation speed. These behavioral tasks, estimating naming and word generation speed, were used as behavioral estimates of language production. Participants also performed the Boston naming task (BNT), which was not analyzed in the current study.

#### 2.2.2 Tasks during MEG measurements

A picture naming task was used to capture the process of language production in the brain. Specifically, we focused on the processing stages prior to overt language production. When studying language production using picture naming, the brain processing also involves visual processing of the to-be-named object. We aimed to eliminate the influence of these processes by contrasting the picture naming task with a baseline visual task (see, e.g.,Price et al., 2005). In the naming task, pictures were presented to the participants, and the participants overtly named the object in the picture after a short delay. If the participant did not know the name of the object, they were instructed to say “skip” (“ohi” in Finnish). In the visual task, participants said “yes” (“kyllä” in Finnish) when a target picture was presented (a red cross in the middle, on top of the picture). The participants also performed other tasks that were not analyzed in the present study (seeAla-Salomäki et al., 2021for details).

#### 2.2.3 Stimuli and procedures

The naming task and the visual task each included 100 different pictures of objects (line drawings). There was no significant difference (Kruskall-Wallis test, p > 0.05) in the naming agreement, word length, or word frequency of the object pictures between the tasks. Half of the pictures (set A) were presented in one measurement session and the other half (set B) in another measurement session. Each picture was presented twice during the measurement session. We aimed to avoid habituation and learning effects across the measurement sessions by using novel stimuli across the measurement sessions (see, e.g.,Nettekoven et al., 2018). Half of the participants started with set A and the other half with set B. The properties of the pictures are described in detail inAla-Salomäki et al. (2021). The visual task, in each measurement session, also included 20 target stimuli not included in the analysis. The picture presentation protocol was the same in both tasks and measurement sessions. Pictures were presented to the participants in blocks, and only one task was performed during one block. Each block contained ten pictures. For the visual task, each block included 1–3 target stimuli. In the block, first a fixation cross appeared on the screen for 1 s, followed by the picture for 300 ms. Then, a blank screen was presented for 1 s, followed by a question mark for 2 s. Participants gave their answer when the question mark was presented. The response was given in a delayed fashion, since we aimed to avoid muscle artifacts related to speech. The task procedure is illustrated inFigure 1. A measurement session included 10 blocks for the naming and 12 blocks for the visual task. The order of the blocks was randomized.

**Fig. 1.**
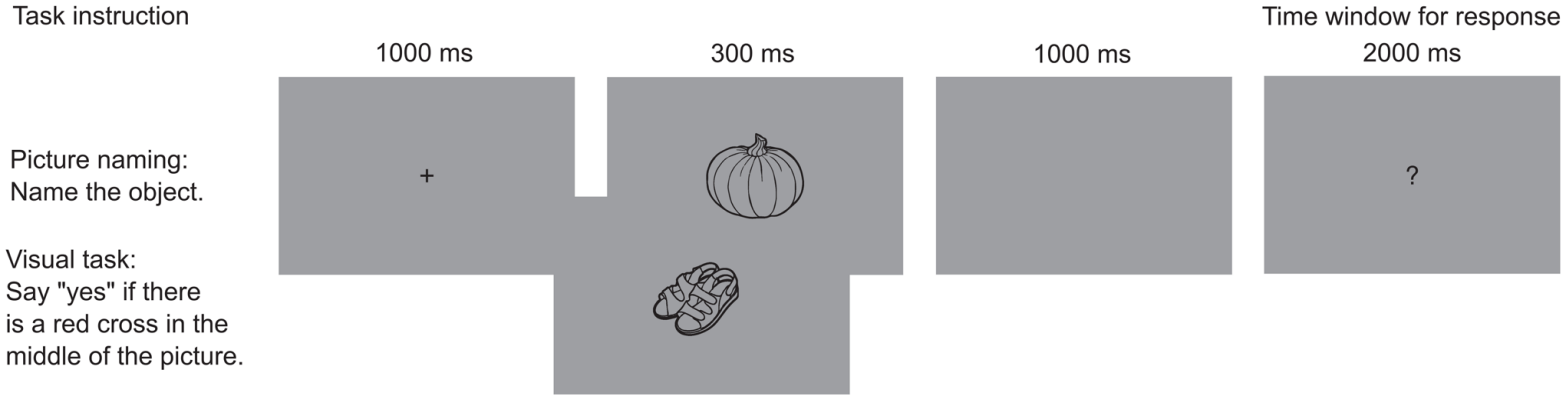
Task procedure. The picture presentation protocol for the picture naming and visual task. Pictures of the illustrated objects from: Papunet, papunet.net, Elina Vanninen, modified versions of the original figures.

### 2.3 MEG and (diffusion) MRI recordings

MEG data were measured at the Aalto NeuroImaging MEG Core (Aalto University, Espoo, Finland) using a Vectorview whole-head MEG device (MEGIN, Helsinki, Finland). The device comprises 306 sensors: 204 planar gradiometers and 102 magnetometers, each coupled to a Superconducting Quantum Interference Device (SQUID) in a helmet-shaped array. Electro-oculogram signals were recorded for identifying eye blinks and saccades. The MEG signals were filtered at 0.1–330 Hz and sampled at 1000 Hz. Details on the MEG recordings are described inAla-Salomäki et al. (2021). MRIs were acquired at the Aalto NeuroImaging Advanced Magnetic Imaging Centre using a Siemens Magnetom Skyra 3.0 T MRI scanner. T1-weighted images were acquired with a standard gradient echo sequence and 1 mm × 1 mm × 1 mm resolution. Diffusion MRI data were acquired with a voxel size of 2 mm × 2 mm × 2 mm in 64 uniformly distributed gradient orientations and a diffusion weighting of b = 1000 s/mm^2^. A total of five b = 0 s/mm^2^images were acquired at the beginning and end of the sequence. The full acquisition was repeated in the reverse phase-encoding direction, resulting in a total of 138 volumes.

### 2.4 MEG preprocessing and connectivity analysis

The spatiotemporal signal space separation method (Taulu & Simola, 2006) was applied to MEG data for noise reduction (16 s temporal window, a subspace correlation limit of 0.98, inside expansion order of 8, and outside expansion order of 3). The head position data were used to transform the MEG data to a common position (MEGIN Maxfilter software package, 2.2.12). Independent Component Analysis was applied to remove eye-blink artifacts. In the naming task, only epochs with correct responses were analyzed (the number of epochs varied from 71 to 98 per participant and measurement).

For estimating all-to-all MEG functional connectivity, we applied a pipeline (van Vliet et al., 2018) that uses coherence as the connectivity metric. The pipeline utilizes the Dynamic Imaging of Coherent Sources (DICS) spatial filter originally proposed byGross et al. (2001)and combines it with a wavelet approach to achieve a high temporal resolution (Kujala et al., 2012;Laaksonen et al., 2008). Connectivity was analyzed for the naming and the visual task in multiple frequency bands and time windows, separately for the two measurement sessions. Data were analyzed in the following frequency bands of interest: 4–7 Hz (theta), 8–13 Hz (alpha), 14–20 Hz (low beta), 21–30 Hz (high beta), 31–45 Hz (low gamma), and 60–90 Hz (high gamma), similarly toAla-Salomäki et al. (2021). Time windows 0–400 ms, 400–800 ms, and 800–1200 ms time-locked to stimulus presentation were selected similarly toAla-Salomäki et al. (2021).

Cross-spectral density matrices (CSDs) across planar gradiometer sensors were calculated for the two tasks in the selected time windows using time-frequency representation (Morlet wavelets) in the frequency range 4–90 Hz, at 1-Hz intervals for the 4–13 Hz frequency range and 2-Hz intervals for the 14–90 Hz frequency range. CSD matrices represent the frequency-domain covariance between the activity recorded at different sensors and they were transformed into a cortical representation using the spatial filter.

For source modeling, a spatial filter (DICS) utilizing individual MRIs was constructed. A surface-based source space in the FreeSurfer (Dale et al., 1999) template brain (“fsaverage”) was created, and these source locations were transformed to each individual subject. We used a regularly spaced grid of 5124 points on each hemisphere, with an average distance of 2.6 mm between neighboring points from which we excluded points that were further than 7 cm from the closest MEG sensor in an example subject. Coherence was estimated between each anatomical grid point and all other grid points, using spherical tangential source orientations that yielded maximum coherence. The coherence was estimated separately for each measurement session, task, frequency band, and time window of interest. To avoid spurious connectivity between closely situated areas due to spatial leakage (field spread), a minimum distance limit of 4 cm between the connection start and end points was applied, and coherence values were always evaluated by contrasting two experimental conditions to cancel out spatial leakage that is constant across conditions (see alsosection 2.6). For more details, seevan Vliet et al. (2018).

### 2.5 Parcellation

For further analysis of the connectivity data, a custom-made parcellation based on the Destrieux FreeSurfer template parcellation for “fsaverage” was used. The custom-made parcellation was constructed to produce as similar-sized parcels as possible to overcome the need of degree correction, while preserving coarse anatomical boundaries when possible. The parcellation included 55 brain regions per hemisphere (seeFig. 2). The details of the parcellation are described inAla-Salomäki et al. (2021). For graph-theoretical analyses of diffusion MRI data, subcortical structures segmented with FIRST in FMRIB Software Library (FSL) were included (Jenkinson et al., 2012).

**Fig. 2.**
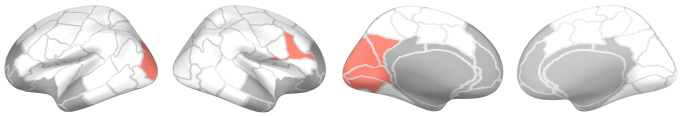
Parcellation and power differences between the tasks. Parcels that were used in the analysis. Gray parcels represent the anterior and subcortical parcels that were excluded from the analysis. Red parcels represent the parcels in which the power difference between the naming and the visual task was larger than 20 % for more than four subjects in individual time-frequency bands and measurement sessions.

### 2.6 Power difference between the tasks

Power differences between the experimental tasks can introduce spurious differences in the coherence estimates (Gross et al., 2013;Kujala et al., 2007,^2008^). Therefore, power levels were computed from the CSD matrices described above for the tasks, frequency bands, and time windows of interest, separately for the two measurement sessions. The power differences were further computed between the naming and the visual task at the parcel level for each subject. We determined the parcels where the power difference between the tasks was larger than 20 % (Schoffelen & Gross, 2009,^2011^) for more than four subjects (Fig. 2). Power differences were detected in only a few parcels (mainly non-overlapping frequency bands and time windows) and were not present in both measurement sessions. Therefore, we considered that the data did not show potentially confounding power differences, and no frequency band was excluded from the further analysis.

### 2.7 Diffusion MRI preprocessing and connectivity analysis

Diffusion MRI data were corrected for subject motion (Leemans & Jones, 2009), eddy current-induced distortions (Andersson & Sotiropoulos, 2016), and susceptibility-induced distortions by using FSL (Jenkinson et al., 2012). Correction for B1 field inhomogeneities was performed using N4BiasFieldCorrection in Advanced Normalization Tools (Avants et al., 2009;Tustison et al., 2010) and MRtrix3 (Tournier et al., 2019). Statistical parametric mapping was used to rigidly coregister the T1-weighted images to the corrected diffusion MRI data (Collignon et al., 1995;Penny et al., 2011).

We used Constrained Spherical Deconvolution to estimate voxel-level fiber orientation distributions with maximum 8th-order spherical harmonics in MRtrix3 (Tournier et al., 2007,^2019^). We performed whole-brain probabilistic streamlines tractography with the iFOD2 algorithm by seeding from the gray matter–white matter interface and using anatomically-constrained tractography to ensure anatomical validity of the streamlines (Smith et al., 2012). Spherical-deconvolution informed filtering of tractograms was used to correct for the density bias in the reconstructed whole-brain streamlines by iteratively optimizing the streamline weights to match the local fiber orientation distributions (Smith et al., 2013,^2015^). Connectivity matrices were weighted by the sum of streamline weights connecting a pair of gray matter regions. Previously, the test-retest reliability of the structural brain connectivity networks has been shown to be high (Roine et al., 2019). The shortest path length between a pair of nodes and edge betweenness centrality was calculated using the Brain Connectivity Toolbox (Rubinov & Sporns, 2010).

### 2.8 Consistency (reliability) analysis of MEG connectivity

#### 2.8.1 Selecting parcel-to-parcel connections to be included in the consistency analysis

As the functional connections related to language processing remain largely unknown, we utilized the measured data to select the connections for the consistency analysis. We evaluated the connectivity modulations between the naming and the visual task in the first measurement session. We conducted paired t-tests for all connections at two significance levels (p < 0.001; uncorrected and p < 0.0001; uncorrected) for the selected frequency bands and time windows. The selection was done at two significance levels, since we wanted to examine how the selection criteria affected on the results. From now on, we refer to the two selection criteria as selection A (p < 0.001; uncorrected) and selection B (p < 0.0001; uncorrected). We did not include the most anterior brain areas (see gray areas inFig. 2) in the analysis, as signals and, subsequently, connectivity of those areas could represent artifactual activity related to eye movements. Even though eye movement artifacts were cleaned from the data, the sub-threshold differences in eye movements between the conditions and individuals could have biased the connectivity estimates of those regions. Additionally, the MEG coverage of the most anterior areas is quite poor as very few sensors are close to those areas, making connectivity estimates less specific for those regions. We thresholded the connectivity results using clustering, such that significant connections were grouped into bundles. Bundles with a minimum of 20 connections and a maximum distance of 1.3 cm between connections were retained. Then, we defined these significant naming-related connections at the parcel level. The resulting parcel-to-parcel connections (all possible connections within parcels) for each frequency band and time window served as input for the subsequent consistency analysis. The modulation direction of connectivity (increase or decrease) was determined by taking the group mean of the connectivity difference between the naming and the visual task during the first measurement session at the parcel level (all possible connections within parcels).

#### 2.8.2 ICC

To establish naming-related connections that were reliable, more specifically consistent, across measurement sessions, we performed intraclass correlation coefficient (ICC) analysis (Shrout & Fleiss, 1979) on the predefined parcel-to-parcel connections, that is, the separate groups of connections per time window and frequency band for selections A and B (seesection 2.8.1). To compute the ICC value for a specific connection at the parcel level, each subject’s connectivity difference between the naming and the visual task was taken and then summed at the parcel level (all possible connections within parcels) separately for the two measurement sessions.

ICC(3,1) refers to consistent agreement and it is defined as:

ICC(3,1)=(BMS−EMS)/(BMS+(k−1)* EMS),

where BMS = between-subjects mean square, EMS = error mean square, and k = number of repeated sessions.

With the consistent connections, we refer to connections with ICC > 0.4. With the inconsistent connections, we refer to connections with ICC ≤ 0.4, which is commonly used as a limit to represent poor reliability (Cicchetti, 1994). However, as noted in a meta-analysis of reliability of fMRI functional connectivity this limit is not absolute (Noble et al., 2019). Another guideline proposed byKoo and Li (2016)states that ICC below 0.5 should be considered as poor reliability. Also, this guideline states that 95% confidence intervals of the ICC values should be reported when evaluating ICC values. We replicated some of the analysis using ICC > 0.5 as a threshold for defining a connection as consistent. An overview of the consistency analysis is presented inFigure 3.

**Fig. 3.**
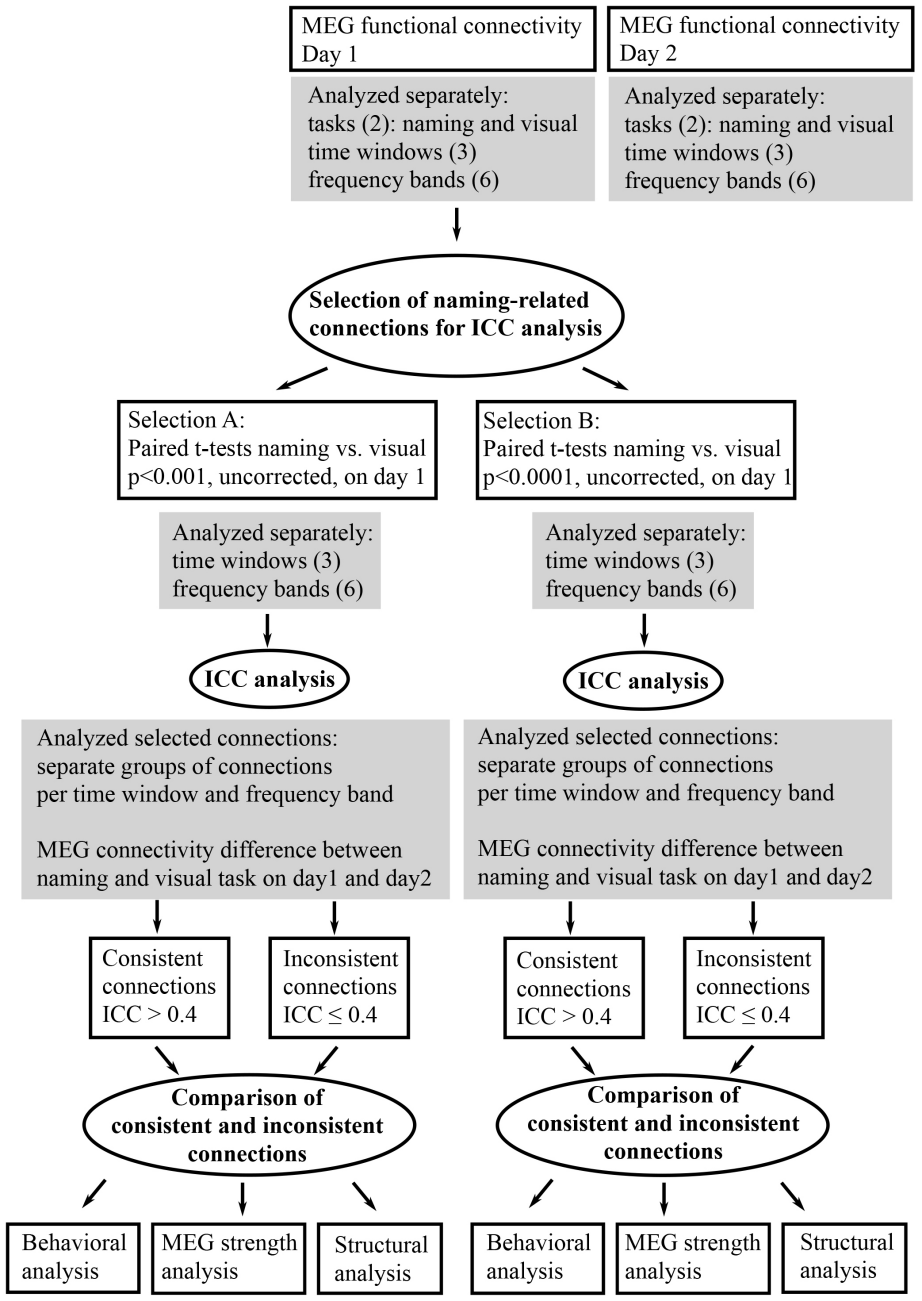
Overview of the consistency analysis. Main analysis steps of the selection of connections for the ICC analysis, ICC analysis, and comparison of consistent and inconsistent connections.

### 2.9 Behavioral, functional, and structural properties of the consistent connections

We studied the properties behind the consistent connections by comparing the consistent connections to the inconsistent ones. For that purpose, connections across all the analysed frequency bands and time windows were pooled together.

#### 2.9.1 Behavioral performance and consistency of connectivity

We studied whether the consistent or inconsistent connections were more frequently associated with language performance. Firstly, we investigated the relationship between behavioral performance and functional connectivity strength. The relationship was investigated at the level of individual connection (connectivity in a certain time window and frequency band). A multiple regression model was built separately for each connection. In the regression model, each subject’s behavioral performances (fluency s, fluency animal, and combined mean variable of RAN and RAS) were set as independent variables. The multiple behavioral measures did not correlate significantly (p > 0.05) with each other. The dependent variables were the MEG connectivity difference, per subject, between the naming and the visual task (mean of both days, connectivity in a certain time window and frequency band). The overall fit of the multiple regression model was tested with an F-test in which the F-statistics represents the proportion of the variance in the dependent variable that can be explained by the independent variables, and the corresponding p-value represents its significance (if p < 0.05, regression model fitted to the data). The number of multiple regression models computed was large and the individual p-values suffer from a multiple comparisons problem (but similarly for the consistent and inconsistent connections which were compared). Secondly, a chi-square test was used to test whether the distribution of connections where the regression model fitted and did not fit to the data was different for the consistent and inconsistent connections.

#### 2.9.2 MEG connectivity strength and consistency of connectivity

We investigated whether the MEG-derived connectivity strength was systematically higher for the consistent or the inconsistent connections. For each connection, each subject’s connectivity difference between the naming and the visual task was obtained and then summed at the parcel level separately for the two measurement sessions, after which the absolute mean across participants and measurement days was calculated. A non-parametric Mann-Whitney U-test was used to test whether the MEG connectivity strength was different between the consistent and the inconsistent connections.

#### 2.9.3 Structural number of direct streamlines, shortest path length, and betweenness centrality related to consistency of functional connectivity

We investigated whether the number of direct streamlines, connection’s shortest path length, and connection’s betweenness centrality differed between the consistent and the inconsistent connections. For each MEG-derived connection, the mean number of direct streamlines, the mean of shortest path length, and the mean of betweenness centrality across participants were calculated. A non-parametric Mann-Whitney U-test was used to test whether these properties were different between the consistent and the inconsistent connections.

## 3 Results

### 3.1 Effect of selection criterion on the number and percentage of consistent connections

**Fig. 4.**
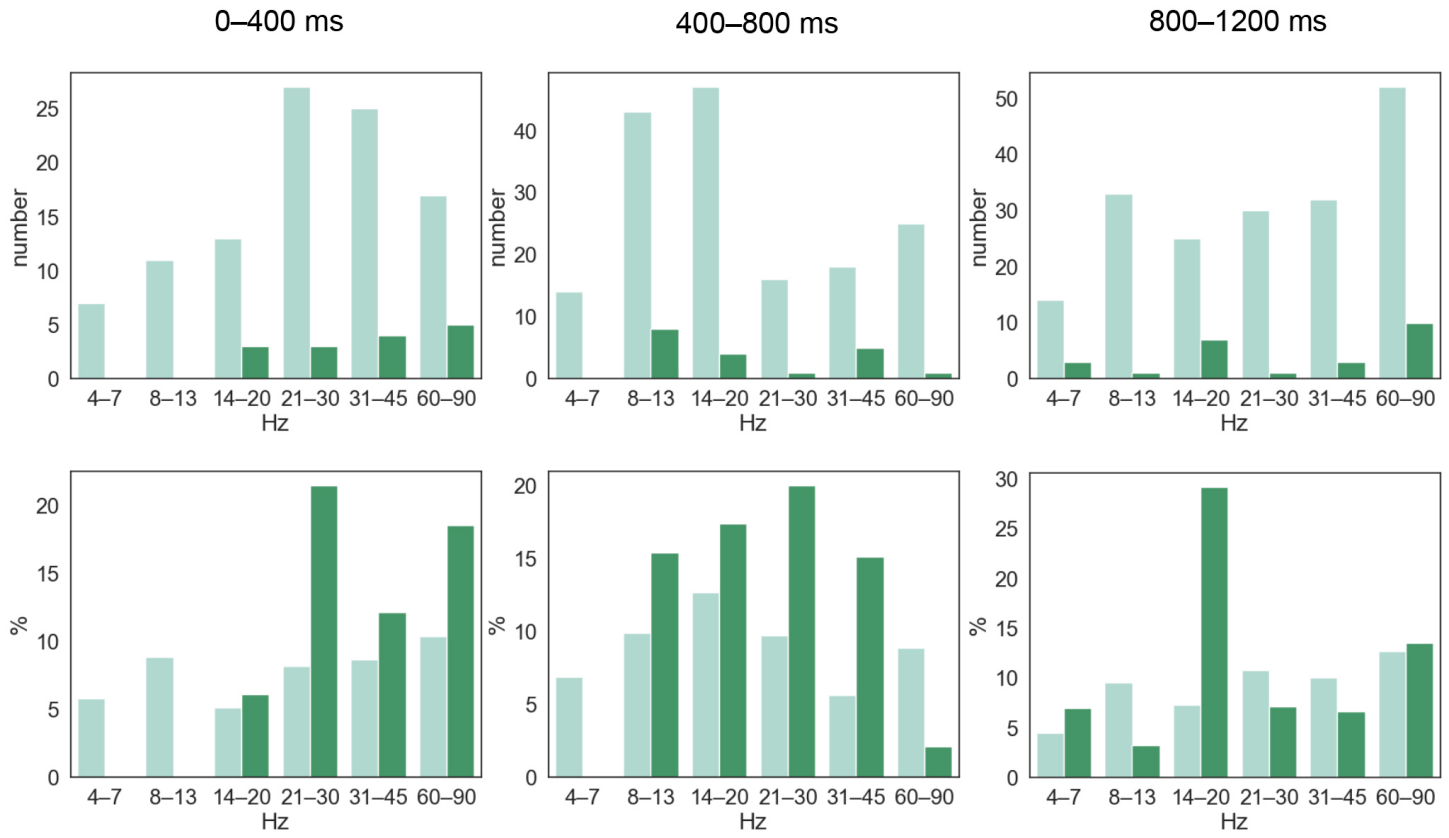
Numbers and percentages of the consistent connections related to picture naming. The numbers of the consistent (ICC > 0.4) connections and the percentages of the consistent connections out of all the connections selected for the ICC analysis separately for each frequency band and time window (after picture onset). Two selection criteria were used to select the connections for the ICC analysis: selection A illustrated in light green color and selection B illustrated in dark green color. Zero means that no connection was selected for the ICC analysis.

To select naming-related connections for ICC analysis, we used two selection criteria (A and B), since we wanted to investigate how the initial choice of connections for consistency analysis affected the results. When pooling together all the frequency bands and time windows, a total of 5054 connections using selection criterion A and 514 connections using selection criterion B were included in the consistency analysis. In the subsequent ICC analysis, 8.9 % (selection A) and 11.5 % (selection B) of those connections were found to be consistent. Overall, the number of consistent connections increased massively using selection A, whereas the percentage of the consistent connections of all the selected connections was a bit lower compared to selection B.Figure 4illustrates how the selection criteria influenced the number and the percentage of the consistent connections in a specific frequency band and time window: the highest percentages of the consistent connections were 12.7 % using selection A and 29.2 % using selection B. As can be observed fromFigure 4, the numbers and percentages of the consistent connections varied across frequency bands and time windows, as well as across the two selection criteria.

### 3.2 Consistent connections related to picture naming

Consistent increased connectivity in the picture naming task compared to the visual task was observed in different time windows, frequency bands, and hemispheres as illustrated inFigure 5. For instance, in the early time window (0–400 ms), a consistent increase in naming-related high gamma connectivity in the visual and posterior temporal regions was detected. From the early (0–400 ms) to the middle (400–800 ms) time window, consistent left-lateralized naming-related high beta connectivity increase was observed mainly in the motor and frontal regions. In the late (800–1200 ms) time window, there was consistent naming-related theta connectivity increase in the motor, frontal, and insular regions. The ICC values and the corresponding 95 % confidence intervals for each connection are presented inSupplementary Table 1. Overall, the consistent naming-related connectivity increases were observed in multiple brain regions, time windows, and frequency bands without a clear emphasis on certain brain region or frequency band.

**Fig. 5.**
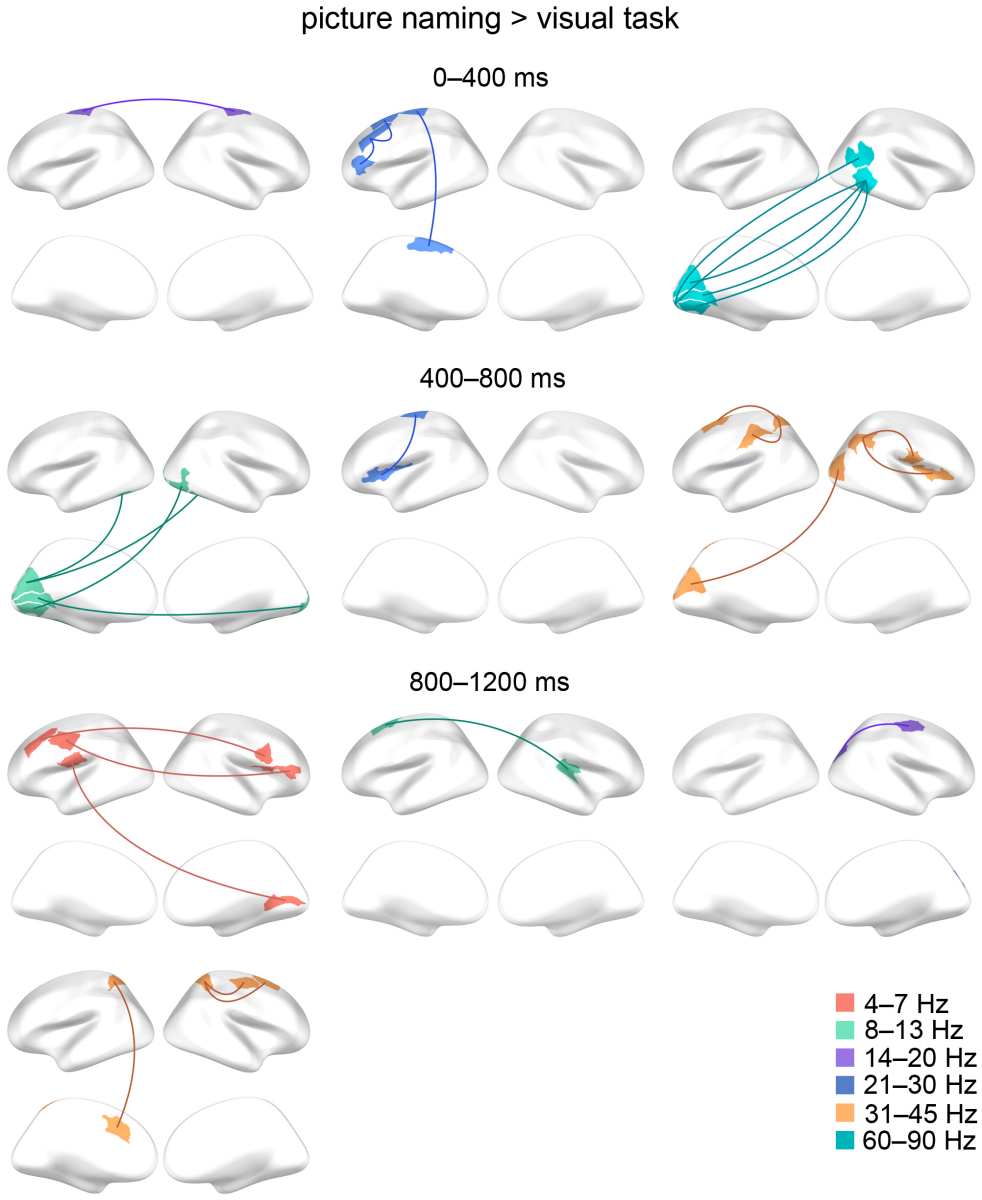
The consistent connections showing increased connectivity in the picture naming versus the visual task. Selection B (picture naming vs. visual task, paired t-tests, p < 0.0001; uncorrected) was used to select the connections for the ICC analysis. The consistent (ICC > 0.4) connections are summarized on schematic brains (upper row lateral and lower row medial views) for different time windows (after picture onset) and frequency bands (illustrated in different colors).

**Fig. 6.**
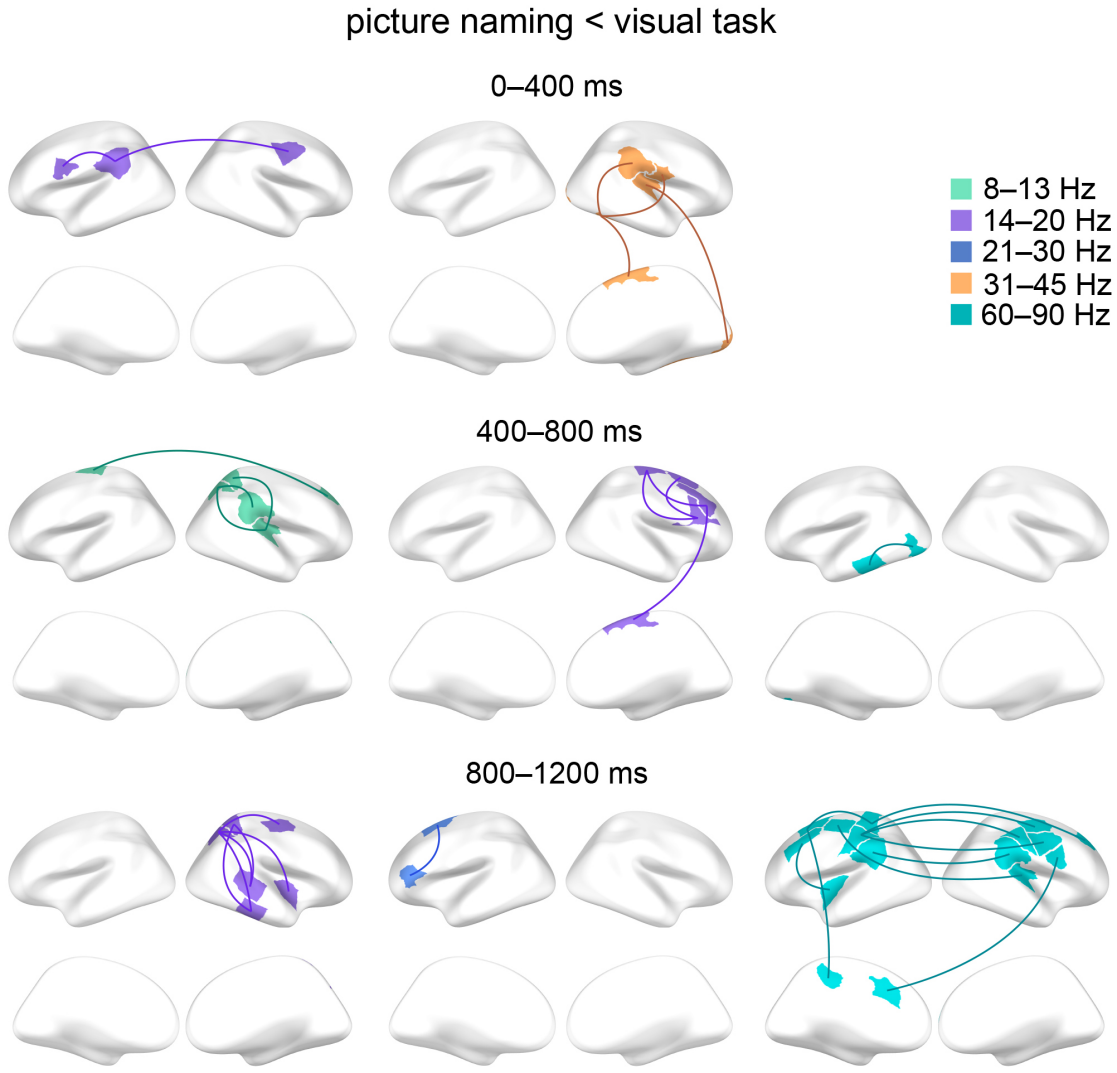
The consistent connections showing decreased connectivity in the picture naming versus the visual task. Selection B (picture naming vs. visual task, paired t-tests, p < 0.0001; uncorrected) was used to select the connections for the ICC analysis. The consistent (ICC > 0.4) connections are summarized on schematic brains (upper row lateral and lower row medial views) for different time windows (after picture onset) and frequency bands (illustrated in different colours).

Figure 6illustrates consistent decreased connectivity in the picture naming task compared to the visual task. A consistent naming-related connectivity decrease was observed most notably in the high gamma band between the bilateral motor areas in the late time window (800–1200 ms). Consistent naming-related connectivity decreases were observed also in other frequency bands and time windows but most notable in the right hemisphere: For instance, alpha connectivity decreased between right parietal and right temporal and parietal regions (400–800 ms). Low beta connectivity decreased mainly between right frontal and right motor areas (400–800 ms) and between right parietal and right temporal regions (800–1200 ms). The ICC values and the corresponding 95 % confidence intervals for each connection are presented inSupplementary Table 2. Overall, the consistent naming-related connectivity decreases were emphasized in bilateral motor areas in the high gamma band and in multiple regions in the right hemisphere in other frequency bands.

**Fig. 7.**
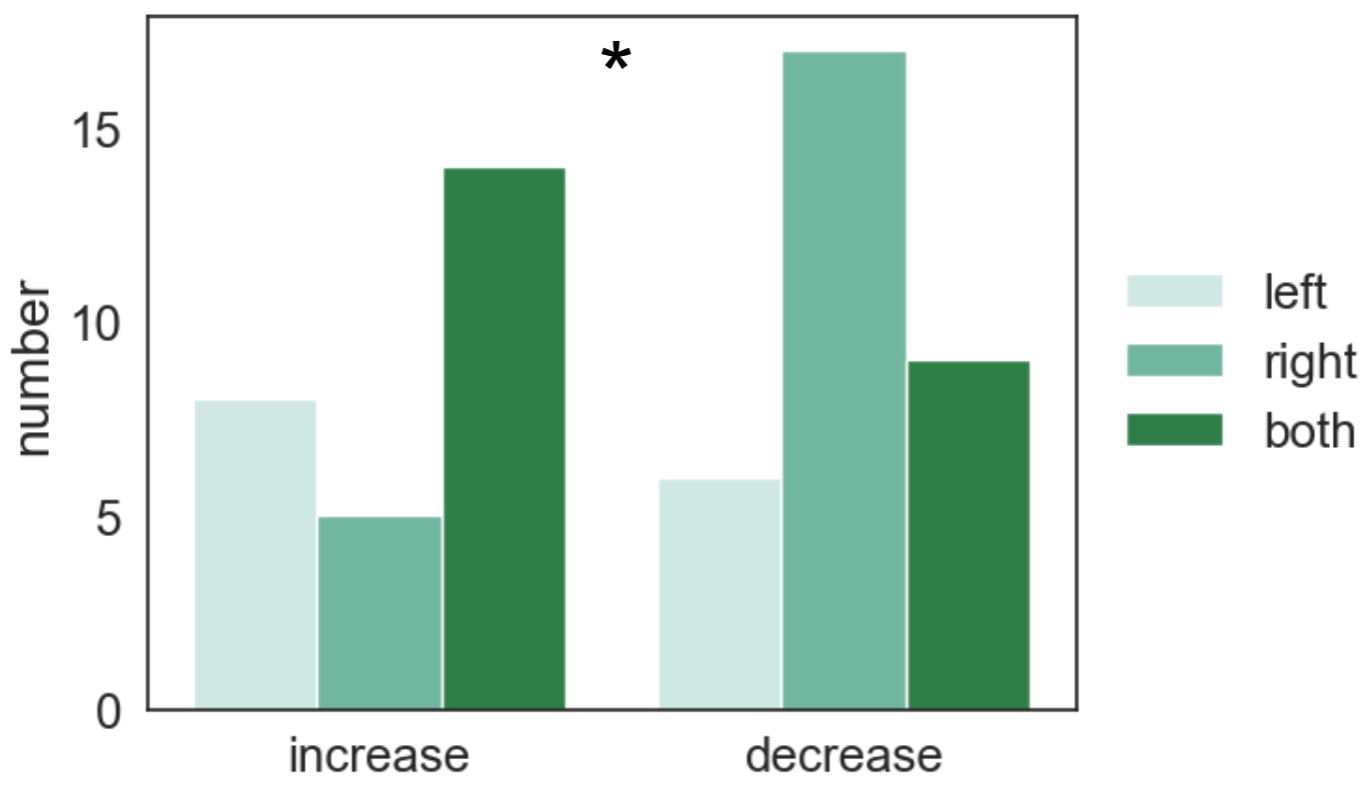
Direction of connectivity modulation and the laterality of the connections. Numbers of consistent naming-related connections (time windows and frequency bands pooled together) grouped by connectivity increase (illustrated inFig. 5) and decrease (illustrated inFig. 6) and whether they were left-lateralized, right-lateralized, or bilateral (both). Relationship between the direction of connectivity modulation and the laterality of the connections was quantified with a chi-square test, *p < 0.05.

Overall, when pooling together time windows and frequency bands, the naming-related consistent connectivity increases (illustrated inFig. 5) were detected in 14 bilateral, 8 left-lateralized, and 5 right-lateralized connections (Fig. 7). Consistent connectivity decreases (illustrated inFig. 6) were detected in 17 right-lateralized, 9 bilateral, and 6 left-lateralized connections (Fig. 7). There was a significant relationship between the laterality of the connections and the direction (increase/decrease) of connectivity modulation (a chi-square test:*X^2^*(2,*N*= 59) = 7.55, p = 0.023).

### 3.3 Language performance and consistency of connections

**Fig. 8.**
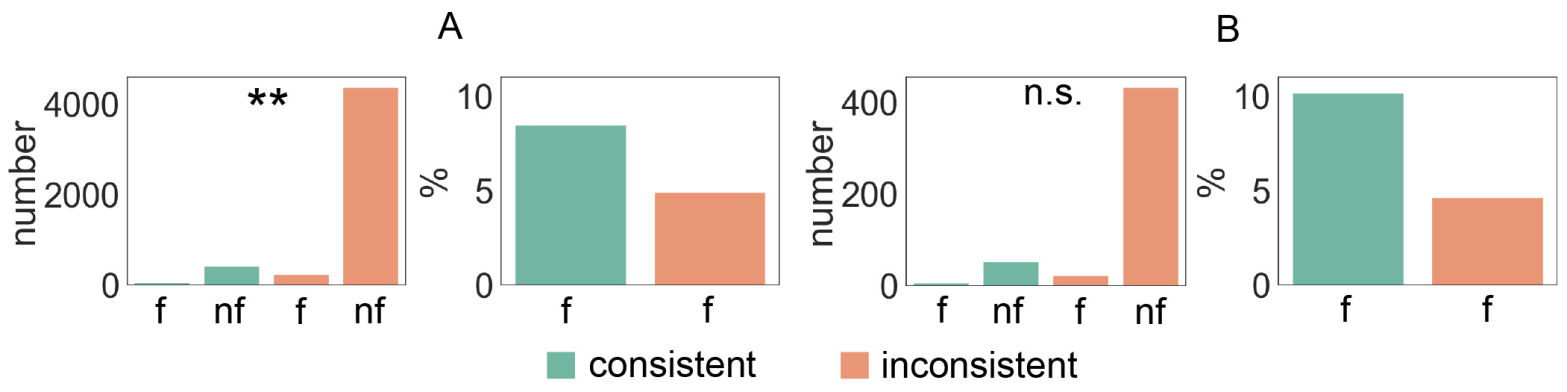
Language performance and consistency of connections. To test a connection’s association with language performance, a multiple regression model was built separately for each connection. The overall fit of the multiple regression model was tested. The number of connections for which the language performance was associated with functional connectivity strength for the consistent and inconsistent connections, divided according to whether the multiple regression model fitted (f) to the data or did not fit (nf) to the data. The percentages of the connections which were associated with language performance (the multiple regression model fitted to the data, f) for the consistent and inconsistent connections. The relationship between consistency of a connection and the connection’s association with language performance was quantified with a chi-square test: n.s.=non-significant, **p < 0.01. Selection A (panel A) and B (panel B) were used to select the connections for the ICC analysis.

The consistent naming-related connections tended to be more frequently associated with language performance (Fig. 8) than the inconsistent connections. When using selection A, there was a significant relationship between consistency of a connection and the connection’s association with language performance (a chi-square test:*X^2^*(1,*N*= 5054) = 9.90, p = 0.0017). A higher proportion of consistent (8.5 %) than inconsistent (4.9 %) connections were associated with language performance. When using selection B, the relationship between consistency of a connection and the connection’s association with language performance was not significant (a chi-square test:*X^2^*(1,*N*= 514) = 2.22, p = 0.14), although a higher proportion of consistent (10.2 %) than inconsistent (4.6 %) connections were again associated with language performance. In conclusion, there was a tendency, irrespective of the selection criterion, that the consistent naming-related connections were more frequently associated with language performance than the inconsistent ones.

### 3.4 MEG functional strength and structural connectivity related to consistency

**Fig. 9.**
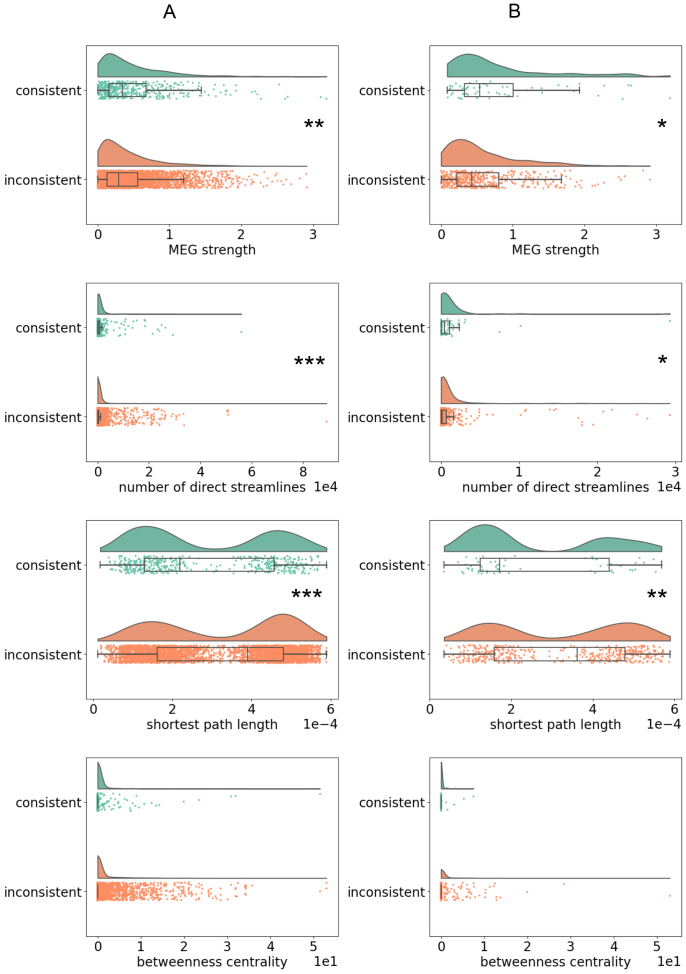
Functional and structural properties of the connections with respect to consistency of connectivity. The distributions, medians, and confidence intervals of the absolute connection strength, number of direct structural streamlines, structural shortest path length, and structural betweenness centrality for the consistent and inconsistent connections. Difference between the consistent and inconsistent connections (Mann-Whitney U-test: *p < 0.05, **p < 0.01, ***p < 0.001). Selection A (panel A) and B (panel B) was used to select the connections for the ICC analysis.

The functional and structural properties underlying the consistent and inconsistent connections are illustrated inFigure 9. The consistent connections were functionally stronger than the inconsistent connections for both selection A and B. Using selection A, the median of connection strength was higher for the consistent (3.4) than for the inconsistent (2.9) connections (Mann-Whitney U-test:*U*= 1124210, p = 0.0022) (Fig. 9A, MEG strength). Using selection B, the median of connection strength was higher for the consistent (5.4) than for the inconsistent (4.3) connections (Mann-Whitney U-test:*U*= 16015, p = 0.016) (Fig. 9B, MEG strength). The consistent connections were structurally stronger than the inconsistent connections for both selection A and B. Using selection A, the median of number of direct streamlines was higher for the consistent (186) than for the inconsistent (84) connections (Mann-Whitney U-test:*U*= 1202460, p = 1.2e-08) (Fig. 9A, number of direct streamlines). Using selection B, the median of number of direct streamlines was higher for the consistent (425) than for the inconsistent (130) connections (Mann-Whitney U-test,*U*= 15867, p = 0.023) (Fig. 9B, number of direct streamlines). The consistent connections were associated with shorter structural path lengths than the inconsistent connections for both selection A and B. Using selection A, the median of shortest path length was lower for the consistent (2.2e-04) than for the inconsistent (3.9e-04) connections (Mann-Whitney U-test:*U*= 884676, p = 4.3e-07) (Fig. 9A, shortest path length). Using selection B, the median of shortest path length was lower for the consistent (1.7e-04) than for the inconsistent (3.6e-04) connections (Mann-Whitney U-test:*U*= 10616, p = 0.0089) (Fig. 9B, shortest path length). The medians of the connections’ structural betweenness centrality were zero both for the consistent and the inconsistent connections using both selection A and B (Fig. 9, betweenness centrality).Supplementary Figure 3provides results of functional and structural properties of the connections with respect to consistency of connectivity when the limit ICC > 0.5 was used to quantify consistent connections. To summarize, irrespective of the selection criterion, the consistent connections were functionally stronger, structurally stronger, and were associated with shorter structural path lengths than the inconsistent connections.

## 4 Discussion

The present MEG study aimed to determine reliable functional connections related to picture naming and define behavioral, functional, and structural properties that may play a role in reliable functional connections. Altogether, we observed that reliable connectivity related to picture naming was modulated in different time windows, frequency bands, and hemispheres as has also been seen in earlier connectivity studies related to language function, with a focus on picture naming (Liljeström et al., 2015), written words (Liljeström et al., 2018), and language comprehension during reading (Schoffelen et al., 2017). The connections that showed reliable naming-related modulation across 2 days formed a fairly small subset of those connections that were initially selected for the consistency analysis and the corresponding ICC values were mostly modest. A particularly salient reliable pattern comprising naming-related high beta connectivity increase in the left motor and frontal regions (0–400 ms and 400–800 ms) and high gamma connectivity decrease in the bilateral motor regions (800–1200 ms) was detected which we interpret to represent the motor preparation of language production. Moreover, we found that the reliable connections, as compared with the non-reliable connections, tended to be more frequently associated with language performance, were functionally and structurally stronger, and were linked with structurally shorter path length.

### 4.1 Reliable connectivity related to picture naming

By focusing on time windows preceding overt speech output in a picture naming task, we here aimed to capture reliable connectivity modulations engaging brain processes related to preparation of language production. In accordance with this aim, we detected a reliable naming-related connectivity pattern comprising high beta connectivity increase in the left motor and frontal regions (0–400 ms and 400–800 ms) which we suggest to represent motor preparation for language production. Previously, connectivity modulation between left motor, premotor, and frontal (Li et al., 2020) regions has been shown to play a role in language production, especially within the beta frequency band (Liljeström et al., 2015). We also detected stronger high gamma connectivity during the visual task than during the naming task in the bilateral motor regions (800–1200 ms). Bilateral motor gamma connectivity during conditions when speech should be suppressed have been detected earlier and suggested to be involved in inhibition of speech production (Liljeström et al., 2015). Here, the observed reliable high gamma connectivity pattern might represent the need to inhibit language production during the visual task.

Among the reliable naming-related connectivity, we also found several connections that may not relate to preparation of language production as such. Any kind of task inevitably also evokes general-level brain processes, not specific to a certain task, such as attention and cognitive control (Fedorenko & Thompson-Schill, 2014). These types of processes may also be represented in the observed reliable connections. Moreover, some of the observed reliable connections might be associated with processing in the baseline visual task. We observed that the reliable naming-related decreases in connectivity, that is, stronger connectivity during the visual task than during the naming task, were highlighted in the right hemisphere. For instance, reliable right-lateralized alpha connectivity decrease between parietal and temporal regions (400–800 ms) and low beta connectivity decrease between parietal and temporal regions (800–1200 ms) were observed. Similarly, an earlier study on semantic judgment and language comprehension reported right-lateralized temporal connectivity patterns, which were proposed to be involved in processes not specific for language (Chai et al., 2016). To summarize, when interpreting the connectivity findings, it is important to keep in mind the multiple factors that cause modulations in connectivity. More specifically, the validity of the observed connectivity findings needs to be considered.

### 4.2 Behavioral performance and reliability

To give additional validation for the observed connectivity findings, this study investigated whether the reliable naming-related connections were more frequently associated with language performance than the non-reliable ones. For language performance, recent studies have emphasized the relevance of resting-state frontal connectivity for word fluency (Mollo et al., 2016), semantic performance (Vatansever et al., 2017), and phonological processing (Yu et al., 2018). In addition, frontal connectivity measured during task states has been linked to reading accuracy and word comprehension (Tomasi & Volkow, 2020). However, there is a lack of research systematically studying the link between the behavioral utility of the connection and its reliability, which was targeted in this study. One earlier resting-state study (Noble et al., 2017) reported that a connection’s reliability was not meaningfully correlated with the connection’s contribution to behavioral prediction, suggesting that the most reliable connections are not the most informative connections for behavior. In contrast, we observed that there was a tendency for reliable naming-related connections to be more frequently associated with language performance. This discrepancy of findings remains to be further investigated in future studies with dedicated experimental designs potentially using a larger sample size. One may ask whether it is meaningful to define reliable connections if those would not have behavioral utility. We suggest that the behavioral utility of the reliable naming-related connections provided additional validation for the findings of the present study.

### 4.3 Functional and structural properties of the connections and reliability

Currently, little is known of the possible factors affecting reliability of functional connectivity. This study investigated the functional properties of the reliable connections. Earlier findings indicate that stronger functional resting state connections are more reliable (Garcés et al., 2016;Noble et al., 2019). Similarly, we observed that the reliable connections were functionally stronger than the non-reliable connections.

The brain’s functional and structural correspondence is an active line of research, especially in network science. Earlier studies have demonstrated similarities in the configuration of structural and functional connectivity, yet the relationship between the two is unknown (see for a reviewAvena-Koenigsberger et al., 2018). Structural connectivity is thought to constrain (Avena-Koenigsberger et al., 2018) and shape (Honey et al., 2010) functional connectivity. For example, the strength of a functional connection between two brain areas depends on the strength of the structural connection between those areas (Hermundstad et al., 2013;Honey et al., 2009). Consequently, structural properties underlying functional connectivity may offer insight about neural communication between brain regions (Avena-Koenigsberger et al., 2018).

Indeed, it is surprising that the brain’s structural connectivity is not commonly utilized when studying the reliability of functional connectivity. This study aimed to diminish this gap by investigating whether the number of direct structural streamlines and the structural length of the connection had an impact on the reliability of the connection. Currently, there is some evidence that structurally connected brain regions have more reliable functional resting-state connections than structurally unconnected brain regions (Griffa et al., 2017;Shen et al., 2015). The results of the present study further demonstrated that the number of direct streamlines were greater for the reliable than the non-reliable connections. Our results also showed that the reliable connections were associated with shorter structural path length than the non-reliable connections. The shortest path length is the topological distance between two brain regions. Minimizing the path length is desirable since longer paths are more susceptible to, for example, noise and longer transmission delays (Avena-Koenigsberger et al., 2018). For these reasons, the shortest paths are thought to ensure reliable and efficient communication between brain regions (Avena-Koenigsberger et al., 2018). We suggest that our finding indicates that the most efficient communication between brain regions is emphasized in reliable functional connections. To conclude, this study offers new information on the importance of the brain’s structural and functional properties in providing insights into what makes a connection reliable.

### 4.4 Methodological considerations and future directions

#### 4.4.1 Coherence as a connectivity measure

In this study, coherence was used as a connectivity measure. Coherence emphasizes the signal transmission along paths that can be reconfigured and is, thus, an excellent connectivity measure to study task-related modulations of connectivity (Fries, 2005;Liljeström et al., 2015;Saarinen et al., 2015). However, coherence is prone to field spread effects, which has to be taken into account. Some other connectivity measures, such as imaginary coherence, are less sensitive to field spread. Nevertheless, the use of imaginary coherence to study task-related modulations of connectivity can be problematic, since it lacks straightforward means to interpret the modulations between tasks (Gross et al., 2013). To summarize, the connectivity results are dependent on the chosen connectivity measure and every measure has advantages and disadvantages which have to be evaluated and addressed. Here, we chose coherence as a connectivity metric, since it is particularly well-suited to study task-related modulations of connectivity. The best effort was made to minimize the effects of field spread.

#### 4.4.2 The selection of connections for the ICC analysis and the choice of ICC value when quantifying reliability

When determining the reliable connections, the selection of the connections that are being studied has its effect on the results. Since the functional connections related to language processing remain largely unknown, we utilized the connectivity data itself to select the connections for the consistency analysis. This was done by evaluating the connectivity modulations between the naming and the visual task in the first measurement session using paired t-tests at two significance levels (selection A: p < 0.001, uncorrected; selection B: p < 0.0001, uncorrected). Choosing the statistical threshold is not straightforward: any statistical threshold is somewhat arbitrary and can be either too strict or too lenient. Using two statistical thresholds enabled us to investigate how the selection criteria affected the results. Here, the statistical thresholds were not set to address the multiple comparisons problem. Consequently, this testing does not provide information on whether the observed connections were statistically significant. The main focus of the study was to investigate the reliability of these selected connections using consistency analysis and also provide additional validation for the findings by investigating the behavioral relevance of the reliable connections. In the present study, the selection criterion had an impact on the number of the reliable connections but it did not affect the general outcome that the reliable connections were associated with behavioral performance.

In the present study, the most anterior brain regions were excluded from the analysis, since even minor differences in eye movements between the conditions, even after eye movement artifact cleaning, and poorer MEG coverage of these regions could have biased the connectivity estimates from these regions. Therefore, this study lacks the ability of finding possible reliable picture naming related connections of the most anterior brain regions, which is a limitation of the present study.

The chosen criterion of ICC > 0.4 to quantify reliable connections (Cicchetti, 1994) is commonly used when studying the reliability of connectivity findings, although this limit is not absolute (see e.g.,Noble et al., 2019). Guidelines for interpreting ICC values have also been proposed byKoo and Li (2016), stating that 95% confidence intervals of the ICC values should be reported when evaluating ICC values (here reported in theSupplementary Material: Table 1, Fig. 1, Table 2 and Fig. 2). In addition,Koo and Li (2016)state that ICC below 0.5 should be considered as poor reliability. As we replicated the analysis of functional and structural properties of the connections with respect to reliability of connectivity using ICC > 0.5 as a threshold for reliable connections (Supplementary Fig. 3), the general conclusions were similar as using ICC > 0.4 as a threshold. We argue that, irrespective of the connection selection criterion or ICC value criterion, our overall conclusions are generalizable to young healthy adults.

#### 4.4.3 How to improve reliability of functional connectivity?

The results of this study illustrated that the percentage of the reliable connections of all the selected connections is quite modest. This finding is in line with an earlier meta-analysis of 25 fMRI studies showing that, on average, individual connections exhibit low reliability (ICC 0.29) (Noble et al., 2019). This raises a question: What should be done in future connectivity studies to improve reliability? The results of this study suggest that it could be beneficial to utilize behavior-based validation as well as functional and structural information when defining reliable functional connections.

### 4.5 Conclusions

To gain a more accurate and comprehensive view of brain networks, we need to be able to define reliable and task-related brain networks. Overall, this study defined reliable functional connectivity related to language production and also contributed to the understanding of what makes a connection reliable. The findings indicate that reliable functional task-related networks can offer behaviorally meaningful information on brain networks and that they do, to some extent, reflect the underlying structural network.

## Supplementary Material

Supplementary Material

## Data Availability

The raw or processed MEG or MRI data from individual subjects cannot be made openly available, according to the ethical permission and national privacy regulations at the time of the data collection. The derived results that served as input for the figures will be made openly available. Analysis methods used in the study are based on openly available software packages for MEG and MRI analysis, as described insection 2. The DICS beamformer as described invan Vliet et al. (2018)has been incorporated into MNE Python (https://mne.tools) version 0.16 and above, and the methods that were used for analysis of connectivity are available in the ConPy package (https://github.com/AaltoImagingLanguage/conpy). The graph theoretical metrics from diffusion MRI data were calculated with the Brain Connectivity Toolbox (Rubinov & Sporns, 2010).
