# Supplementary material for: Test-retest reliability of MEG functional brain connectivity related to language production: Behavioral, functional, and structural underpinnings of reliable connectivity
Source: Imaging Neurosci (Camb). 2025 Apr 25;3:imag_a_00550. doi: 10.1162/imag_a_00550 (PMC12319785; doi:10.1162/imag_a_00550)
Supplement: Supplementary Material [file imag_a_00550-supp.pdf]

SUPPLEMENTARY MATERIAL

Supplementary Table 1. The ICC values and the corresponding 95 % confidence intervals for selection B. The values correspond to the consistent connections showing increased connectivity in the picture naming vs. the visual task in different time windows (after picture onset) and frequency bands. Connection numbers displayed in Supplementary Figure 1.

Time window (ms)	Frequency (Hz)	Connection number	ICC	95% CI
0–400				
	14–20	1	0.40	[-0.050, 0.72]
	21–30	2	0.48	[0.040, 0.76]
	21–30	3	0.43	[-0.020, 0.73]
	21–30	4	0.42	[-0.030, 0.73]
	60–90	5	0.61	[0.23, 0.83]
	60–90	6	0.55	[0.13, 0.80]
	60–90	7	0.47	[0.030, 0.75]
	60–90	8	0.58	[0.19, 0.82]
	60–90	9	0.52	[0.10, 0.78]
400–800				
	8–13	10	0.42	[-0.030, 0.73]
	8–13	11	0.41	[-0.040, 0.72]
	8–13	12	0.43	[-0.020, 0.73]
	8–13	13	0.54	[0.13, 0.79]
	21–30	14	0.50	[0.070, 0.77]
	31–45	15	0.41	[-0.040, 0.72]
	31–45	16	0.44	[0.00, 0.74]
	31–45	17	0.56	[0.15, 0.80]
	31–45	18	0.55	[0.14, 0.80]
	31–45	19	0.47	[0.040, 0.76]
800–1200				
	4–7	20	0.41	[-0.040, 0.72]
	4–7	21	0.45	[0.010, 0.75]
	4–7	22	0.70	[0.37, 0.87]
	8–13	23	0.42	[-0.030, 0.73]
	14–20	24	0.41	[-0.040, 0.72]
	31–45	25	0.44	[-0.010, 0.74]
	31–45	26	0.44	[0.00, 0.74]
	31–45	27	0.62	[0.24, 0.83]

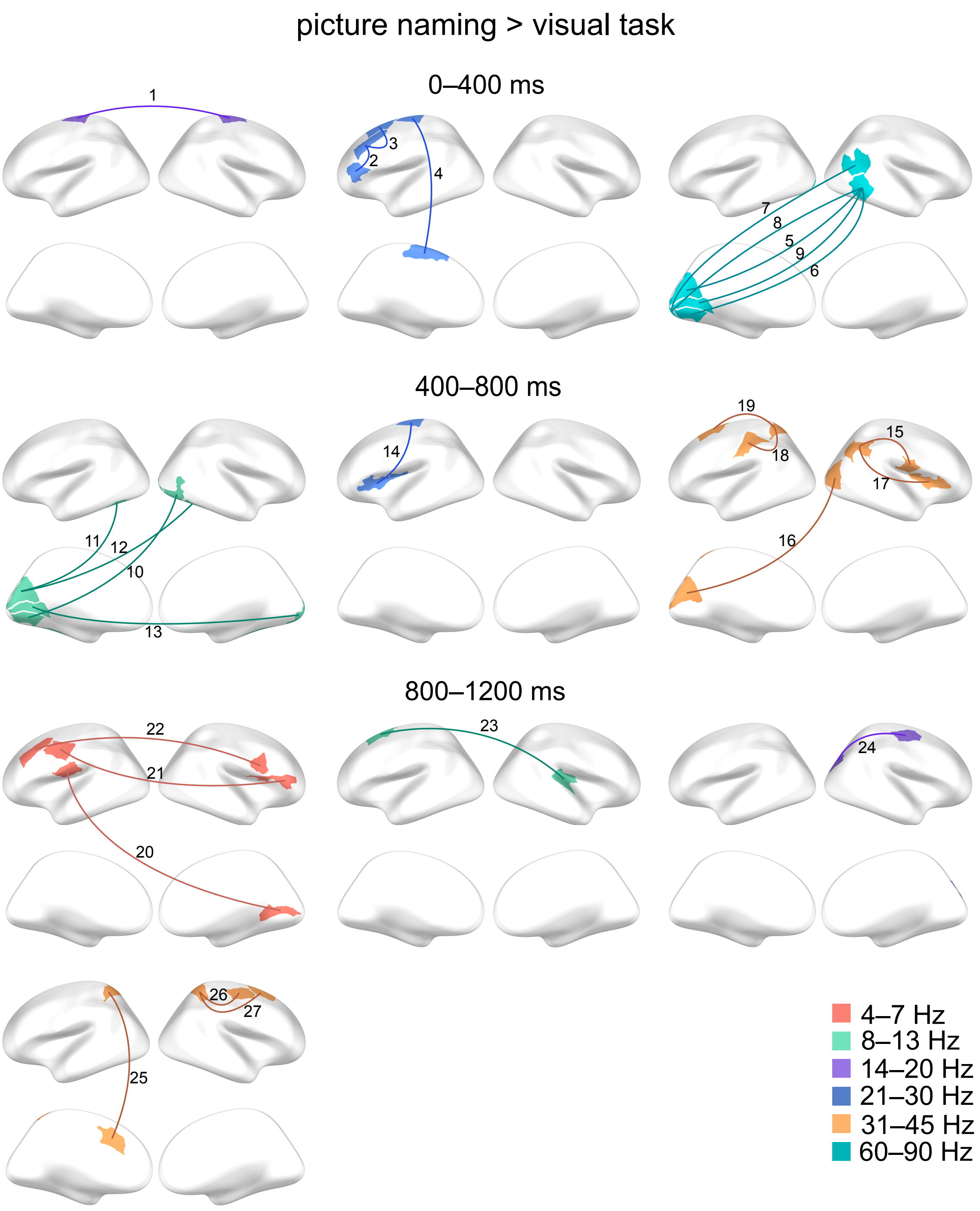

Supplementary Figure 1. Connection numbers. Connection numbers are displayed for Supplementary Table 1.

Supplementary Table 2. The ICC values and the corresponding 95 % confidence intervals for selection B. The values correspond to the consistent connections showing decreased connectivity in the picture naming vs. the visual task in different time windows (after picture onset) and frequency bands. Connection numbers displayed in Supplementary Figure 2.

Time window (ms)	Frequency (Hz)	Connection number	ICC	95% CI
0–400				
	14–20	1	0.45	[0.010, 0.75]
	14–20	2	0.41	[-0.040, 0.73]
	31–45	3	0.68	[0.33, 0.86]
	31–45	4	0.49	[0.060, 0.77]
	31–45	5	0.52	[0.090, 0.78]
	31–45	6	0.43	[-0.020, 0.73]
400–800				
	8–13	7	0.46	[0.02, 0.75]
	8–13	8	0.45	[0.01, 0.75]
	8–13	9	0.55	[0.14, 0.80]
	8–13	10	0.58	[0.18, 0.81]
	14–20	11	0.43	[-0.010, 0.74]
	14–20	12	0.45	[0.010, 0.74]
	14–20	13	0.51	[0.090, 0.78]
	14–20	14	0.54	[0.13, 0.79]
	60–90	15	0.46	[0.020, 0.75]
800–1200				
	14–20	16	0.67	[0.33, 0.86]
	14–20	17	0.71	[0.38, 0.88]
	14–20	18	0.50	[0.070, 0.77]
	14–20	19	0.45	[0.00, 0.74]
	14–20	20	0.42	[-0.020, 0.73]
	14–20	21	0.47	[0.030, 0.76]
	21–30	22	0.47	[0.040, 0.76]
	60–90	23	0.61	[0.23, 0.83]
	60–90	24	0.41	[-0.050, 0.72]
	60–90	25	0.43	[-0.010, 0.74]
	60–90	26	0.40	[-0.050, 0.72]
	60–90	27	0.56	[0.16, 0.80]
	60–90	28	0.60	[0.21, 0.82]
	60–90	29	0.70	[0.32, 0.86]
	60–90	30	0.68	[0.33, 0.86]
	60–90	31	0.43	[-0.010, 0.74]
	60–90	32	0.63	[0.26, 0.84]

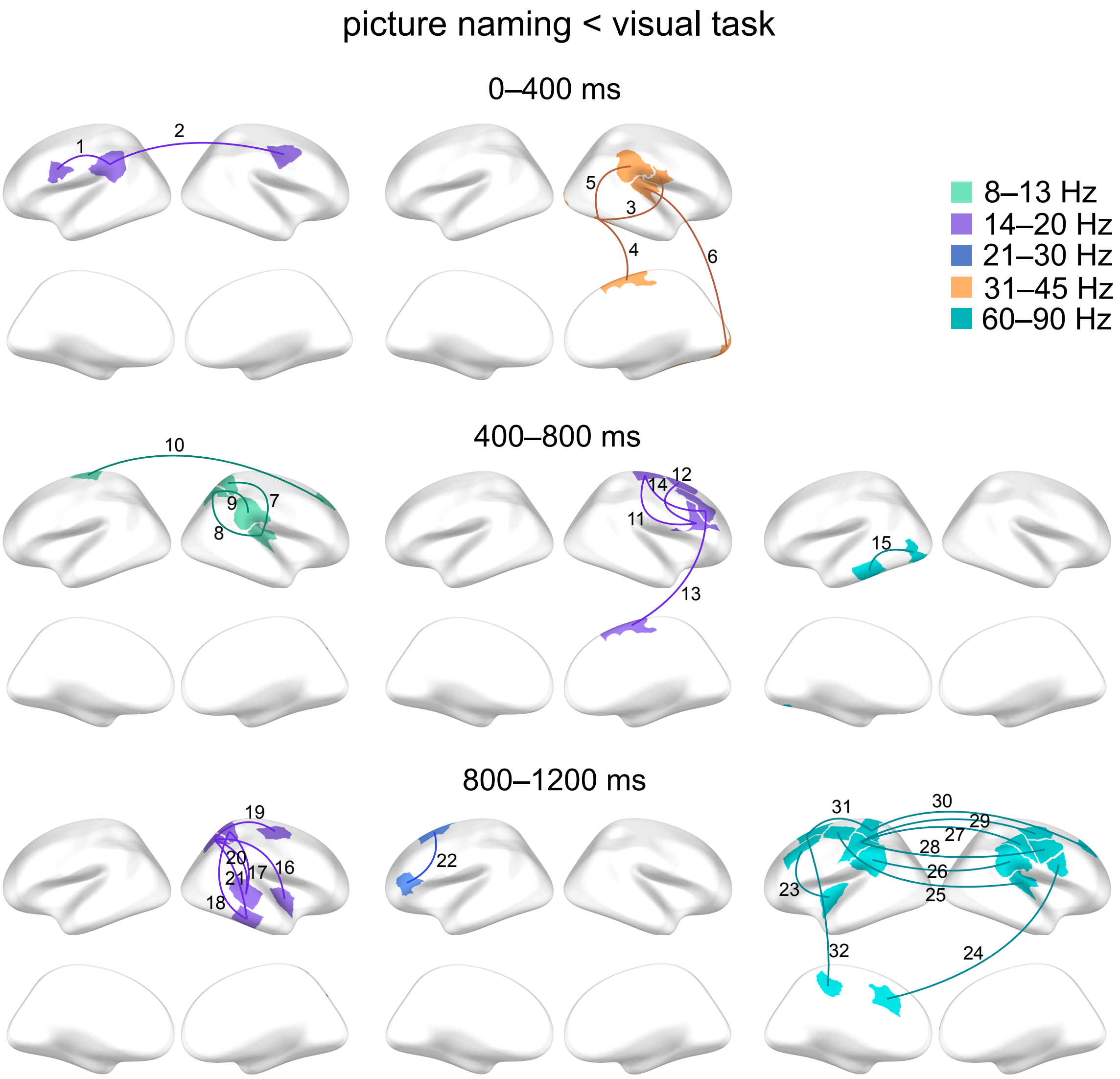

Supplementary Figure 2. Connection numbers. Connection numbers are displayed for Supplementary Table 2.

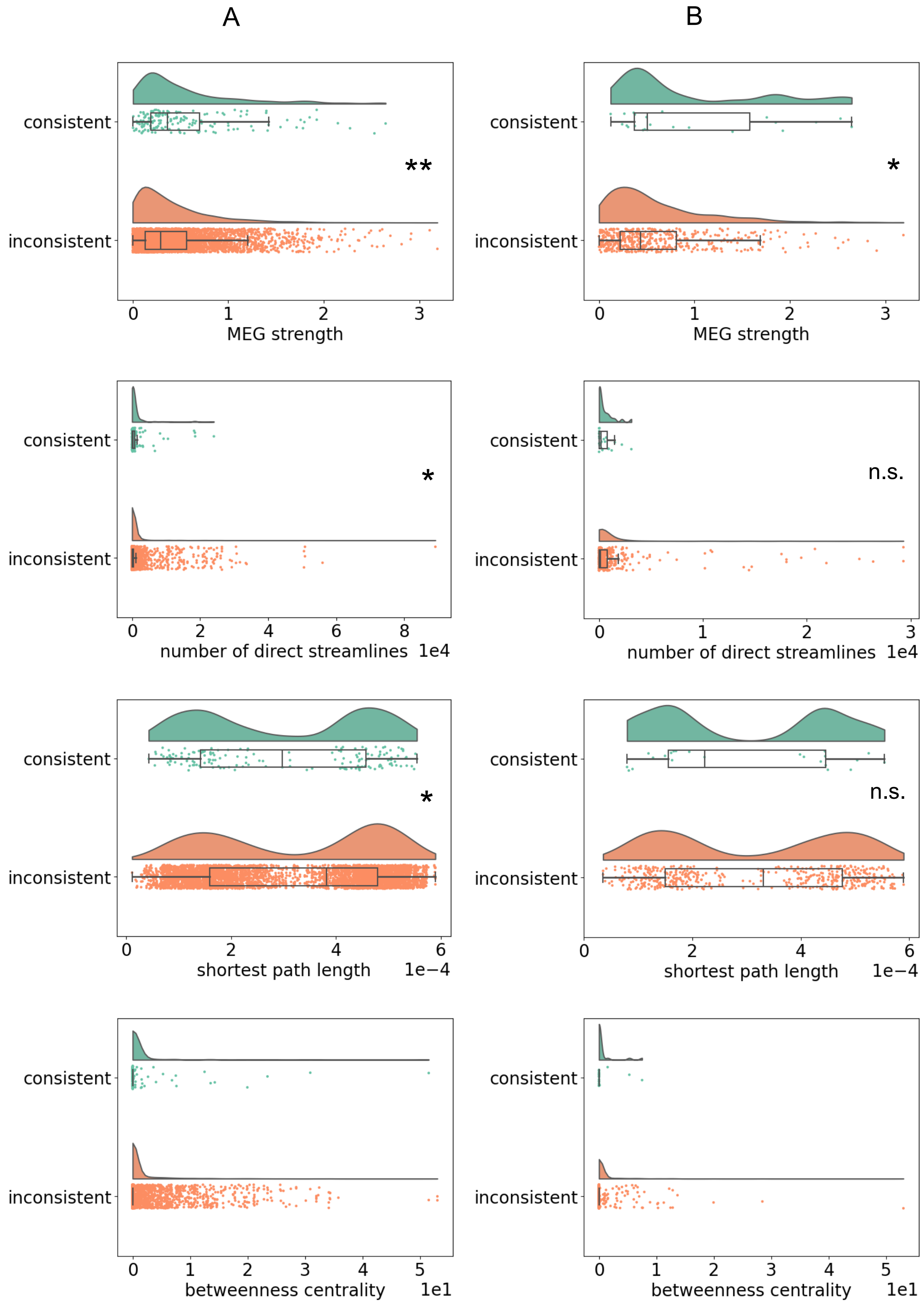

Supplementary Figure 3. Functional and structural properties of the connections with respect to consistency of connectivity using ICC > 0.5 as a threshold for consistent connections. For selection A (A) and B (B), the distributions, medians, and confidence intervals of the absolute MEG strength, number of direct structural streamlines, structural shortest path length, and structural betweenness centrality for the consistent and the inconsistent connections. Mann-Whitney U-test: n.s.=non-significant, *p<0.05, **p<0.01.
